# Comprehensive analysis of mitochondria-related genes indicates that PPP2R2B is a novel biomarker and promotes the progression of bladder cancer via Wnt signaling pathway

**DOI:** 10.1186/s13062-024-00461-6

**Published:** 2024-02-26

**Authors:** Du Shen, Shaosan Kang

**Affiliations:** 1 College of Clinic Medical, North China University of Science and Technology, Tangshan, China; 2 North China of Science and Technology Affiliated Hospital, Tangshan, China

**Keywords:** PPP2R2B, Mitochondrial-related genes, Biomarker, Metastasis, Bladder cancer, Wnt signaling pathway

## Abstract

Bladder cancer (BC) is the fourth and tenth most common malignancy in men and women worldwide, respectively. The complexity of the molecular biological mechanism behind BC is a major contributor to the lack of effective treatment management of the disease. The development and genesis of BC are influenced by mitochondrial retrograde control and mitochondria-nuclear cross-talk. However, the role of mitochondrial-related genes in BC remains unclear. In this study, we analyzed TCGA datasets and identified 752 DE-MRGs in BC samples, including 313 down-regulated MRGs and 439 up-regulated MRGs. Then, the results of machine-learning screened four critical diagnostic genes, including GLRX2, NMT1, PPP2R2B and TRAF3IP3. Moreover, we analyzed their prognostic value and confirmed that only PPP2R2B was associated with clinical prognosis of BC patients and Cox regression assays validated that PPP2R2B expression was a distinct predictor of overall survival in BC patients. Them, we performed RT-PCR and found that PPP2R2B expression was distinctly decreased in BC specimens and cell lines. Functional experiments revealed that overexpression of PPP2R2B distinctly suppressed the proliferation, migration and invasion of BC cells via Wnt signaling pathway. In summary, these research findings offer potential molecular markers for the diagnosis and prognosis of BC, with the discovery of PPP2R2B particularly holding significant biological and clinical significance. This study provides valuable clues for future in-depth investigations into the molecular mechanisms of BC, as well as the development of new diagnostic markers and therapeutic targets.

## Introduction

Bladder cancer (BC) is the 10th most common malignancy worldwide, resulting in about 170,000 deaths worldwide every year [1]. On a global scale, BC exhibits a higher incidence rate, particularly in developed countries, with male patients experiencing a significantly higher incidence than females, showing a gender difference of two to three times [2]. BC predominantly affects individuals in the middle-aged and elderly population, particularly those aged 50 and above, with the elderly facing a comparatively higher risk of developing the disease. Geographical variation is also a noteworthy characteristic in the epidemiology of BC [3, 4]. There are significant differences in incidence rates among different regions, likely attributable to factors such as environmental conditions, lifestyle, and dietary habits. Smoking is identified as a primary risk factor for BC, with smokers facing a significantly higher risk compared to non-smokers. Additionally, exposure to chemicals in the occupational environment, such as benzene and organic solvents, is considered to be associated with the occurrence of BC [5, 6]. Genetic factors may play a certain role in the onset of BC, as evidenced by the presence of clusters of BC in some families, indicating a genetic influence on an individual's susceptibility to BC. Epidemiological data indicates that 5–15% of patients with muscle-invasive bladder cancer face a bleak prognosis and a lower 5-year survival rate due to the disease being already in an advanced stage at the time of diagnosis. Patients dealing with metastasis face a double whammy: early detection is challenging, and there are currently no effective targeted treatments. Therefore, to discover new diagnostic and treatment targets for BC, it is crucial to do additional research into the molecular and biological processes underpinning distant metastasis.

Machine Learning (ML) is a branch of artificial intelligence that focuses on enabling computer systems to perform tasks without explicit programming by learning patterns and rules from data [7]. In the field of medicine, ML is widely applied to disease diagnosis and the optimization of treatment plans, with tumor diagnosis being a crucial application area. In the process of screening tumor diagnostic genes, the first step involves data collection, including gene expression data, protein expression data, and more [8, 9]. Subsequently, data preprocessing is carried out to clean, standardize, and select features to enhance model performance. Appropriate ML models, such as Support Vector Machines, decision trees, random forests, etc., are selected, and a predictive model for tumor diagnostic genes is established through training with labeled training data [10, 11]. After model training, evaluation is conducted using unlabeled test data, and the model is optimized to improve its generalization ability. Ultimately, the trained model is applied to new, unknown samples for the screening of tumor diagnostic genes. In recent years, several studies have reported the important potential of Machine Learning used as novel methods for the identification of novel diagnostic genes in several types of tumors.

Mitochondria are organelles within cells that play a crucial role in energy production. They generate the primary form of cellular energy, adenosine triphosphate (ATP), through the process of oxidative phosphorylation [12–14]. Mitochondria serve as the site for cellular respiration, where organic substances react with oxygen to release energy [15]. Regarding the relationship between mitochondria and tumors, some studies suggest a certain association, although research is ongoing, and scientists are still delving into the specific mechanisms of this relationship [16–18]. Certain research suggests that tumor cells may demonstrate unique mitochondrial functions in comparison to normal cells, encompassing morphological alterations in mitochondria, mutations within mitochondrial DNA, and functional abnormalities. These changes may be related to the survival, proliferation, and anti-apoptotic (anti-cell death) capabilities of tumor cells [19, 20]. On the other hand, mitochondrial dysfunction can lead to disturbances in cellular energy metabolism, which may be one of the reasons for the rapid growth and division of some tumor cells [21, 22]. Additionally, mitochondria are also associated with the process of apoptosis (programmed cell death), and some tumor cells may evade normal cell death mechanisms by modulating the apoptotic signaling pathways of mitochondria. In addition, many mitochondria-related genes (MRGs) have been identified. However, their expression and function in BC remained largely unclear. In this study, we aimed to used Machine Learning to screen critical diagnostic MRGs and further explored their prognostic value and function. Importantly, we identified a diagnostic and prognostic MRG PPP2R2B which promoted the proliferation and metastasis via Wnt/β-catenin signaling.

## Materials and methods

### TCGA-BLCA cohort and GEO cohort

The Cancer Genome Atlas (TCGA) data portal (https://gdc-portal.nci.nih.gov/) was used to get the level-three transcriptome RNA sequencing data and the associated clinicopathological features of patients with BC. Moreover, Datasets GSE13507 and GSE3167 were used to extract RNA-sequence information from both normal and BC samples. Furthermore, we used MSigDB (https://www.gsea-msigdb.org/gsea/msigdb) to screen 1513 MRGs.

### Differential expression analysis

For 1513 MRGs found in both normal and cancerous tissues, we used the GSE13507 database to get their expression profiles. Then, we used the student's t-test in R to find differentially expressed MRGs (DE-MRGs) between the two sets of data. For statistical significance, a *p*-value below 0.05 was considered.

### GO (Gene Ontology) and KEGG (Kyoto Encyclopedia of Genes and Genomes) pathway analyses

GO is a standardized biological terminology system used to describe the biological functions of genes and proteins. It categorizes biological functions into three main levels: Molecular Function, Cellular Component, and Biological Process. KEGG is a comprehensive database designed to integrate information about genomes, chemical substances, and biological systems. It offers in-depth information on a variety of topics, including illnesses, metabolic pathways, gene functions, and biological pathways, among other things. ClusterProfiler is a R program that is widely utilized in the field of bioinformatics [23]. Its primary purpose is to perform functional enrichment analysis on high-throughput biological data. It is primarily used to analyze gene sets, revealing the enrichment of these genes in biological functions, pathways, and metabolic pathways, aiding researchers in gaining a deeper understanding of experimental results. The clusterProfiler (version 3.10.1) software was utilized in order to carry out the O and KEGG analyses. DOSE (Disease and Ontology Semantic and Enrichment) is an R package specifically designed for disease-related functional enrichment analysis in bioinformatics. Its primary function is to assist researchers in understanding the association between gene sets and diseases, as well as the enrichment of these genes in biological processes, cellular components, and molecular functions. To aid with interpretation, the enrichplot and DOSE programs were utilized to visualize the enrichment results. The threshold values used were *p* < 0.05 and adjusted *p* < 0.05.

### Screening of candidate diagnostic biomarkers

In this study, the support vector machine recursive feature elimination (SVM-RFE) and least absolute shrinkage and selection operator (LASSO) were employed to identify noteworthy diagnostic genes. SVM-RFE is a feature selection method based on Support Vector Machine (SVM) [24]. It combines the classification performance of SVM with the idea of recursive feature elimination to help identify the critical diagnostic genes. SVM-RFE considers the interactions between features during the feature selection process, focusing not only on the individual contribution of each feature but also on their combined effects. Genes with greater discriminatory power were located using the SVM-RFE technique. LASSO is a regularization method used for linear regression and related models. It introduces sparsity by adding an L1 regularization term to the loss function of linear models, aiding in the selection of the most important features and pushing the coefficients of other features toward zero [25]. LASSO is a powerful regularization technique that not only reduces model complexity and prevents overfitting but also facilitates feature selection in regression analysis, enhancing the interpretability and generalization performance of the model. LASSO was performed using the “glmnet” package in R. Applying two algorithms to the metadata cohort and using the GSE13507 dataset to conduct an analysis of genes that overlapped between the two methods was done with the intention of further evaluating the expression levels of potential diagnostic biomarkers.

### Construction of the nomogram

A Nomogram is a graphical tool used to visually represent and calculate the impact of various factors in a model on a specific outcome. It is an intuitive tool commonly employed in the fields of medicine and statistics to assist researchers and clinicians in better understanding and applying complex predictive models. Through the use of the rms R package (https://cran.r-project.org/web/packages/rms/), all of the prognostic factors were utilized in the development of a prognostic nomogram. This nomogram was used to evaluate the probability of 0.5-, 1-, and 3-year overall survival for patients with BC. The C-index and the area under the curve (AUC) were utilized in order to objectively evaluate the discrimination performance of the nomogram. In order to graphically test the nomogram's capacity to differentiate across different categories, calibration plots were also utilized.

### Construction of the prognostic signature

In order to determine the predictive power of MRGs, the LASSO Cox regression model was used to incorporate all of the genes. Next, we determined the risk score for each and every patient with BC. The risk score formula was as follows: Risk Score = ∑Coefi * Xi (Coefi: the regression coefficient, Xi: gene expression value). Following the clustering of BC patients in the TCGA database into two groups (low-risk and high-risk) according to the median risk score, Kaplan–Meier analysis was employed in order to examine the overall survival rates of the two risk groups.

### Patients and specimens

Between 2021 and 2022, 10 pairs of BC tissues and adjacent normal tissues were collected from 10 patients during radical cystectomy. Each patient has previously signed an informed consent. This study has been approved by the ethics committee institution of North China of Science and Technology Affiliated Hospital.

### Cell culture and transfection reagents

We obtained human BC cell lines 5637, RT4, J82, T24, and SW780 from the American Type Culture Collection (ATCC, Manassas, Virginia, United States of America). Additionally, we obtained human SV40 immortalized uroepithelium cell SVHUC-1 (CRL-9520). The cells were kept in Dulbecco's modified Eagle medium, which contained 10% fetal bovine serum (provided by Thermo Fisher Scientific Inc., Waltham, Massachusetts, United States), at a temperature of 37 degrees Celsius and a carbon dioxide concentration of 5%. There have been claims made by vendors that mycoplasma contamination tests have been performed on cells on a regular basis. Control vector and over-expression vector harboring full length cDNA of PPP2R2B were purchased from Jikai Gene (Shanghai, China).

### Quantitative reverse transcription polymerase chain reaction (RT-qPCR) analysis

For the purpose of collecting total RNA, the TRIzol Reagent (Invitrogen) was utilized. Following that, cDNAs were produced by reverse transcription from RNAs using M-MLV reverse transcriptase, which was manufactured by Promega in Madison, United States of America. For the purpose of analyzing RNA expression, the SYBR Green PCR Master Mix (Applied Biosystems, Foster City, California, United States) was utilized. The normalization of gene expression was dependent on GAPDH, which was determined using the 2 − ΔΔCt protocol. The independent assay was carried out in triplicate. RT-PCR for PPP2R2B was carried out with the forward primer 5’- CCACACGGGAGAATTACTAGCG-3’ and reverse primer 5’- TGTATTCACCCCTACGATGAACC-3’. GAPDH forward: 5’- ACAACTTTGGTATCGTGGAAGG-3’ and reverse: 5’- GCCATCACGCCACAGTTTC -3′.

### Cell counting Kit-8 assay

The initial step involved the seeding of the cells into 96-well culture plates, with each well containing a density of 5 × 10^3^ cells. After that, the plates were placed in an incubator for a period of time ranging from 24 to 48 to 72 h. Cell Counting Kit-8 (CCK-8) solution (Beyotime; Cat: C0038) is currently available. For a duration of one hour, 10 μL/well was added and cultured in an incubator. In order to have an accurate representation of the cell proliferation activity, the absorbance of each well was measured using a plate reader at a wavelength of 450 nm.

### TUNEL staining

During the night, the cells were cultivated. After being raised twice with PBS, the cells were fixed with 4% paraformaldehyde for fifteen minutes, and then they were permeabilized with 0.25% Triton-X 100 for twenty minutes. This process was repeated until the cells were completely permeabilized. Procedures for TUNEL assays were carried out in accordance with the guidelines provided by Roche, the manufacturer. To summarize, the cells were originally treated with the Click-iT reaction cocktail after being incubated for 45 min at 37 degrees Celsius in a terminal dexynucleotidyl transferase (TdT) reaction cocktail during the initial phase of the experiment. This was done in order to ensure that the cells maintained their integrity. In order to stain the nucleus, it was decided that either hematoxylin or methyl green would be acceptable.

### Colony formation assays

Assays for colony formation were carried out in order to assess the growth of the cells. To summarize, a 6-well plate was planted with 400 transfected RT4 and J82 cells as the starting point. Additionally, cells were kept alive until colonies were established. After that, the colonies were dyed with crystal violet and fixed with paraformaldehyde for a period of thirty minutes. After then, the colonies were computed for purpose of further investigation.

### Wound-healing scratch assay

For the purpose of determining the capacity for cellular migration, the wound-healing scratch assay was utilized. The cells were planted in a plate with six wells one day before the experiment. Scratching the cells with a 20 μl pipette tip was followed by incubation of the cells in a medium that did not include any serum for a period of 72 h. At 24, 48, and 72 h, scratch wounds were observed and photographed for documentation purposes. Image J software was utilized in order to accomplish the task of measuring the distance between the two edges of the scratch wound.

### Transwell assay

RT4 and J82 cells that had been transfected were resuspended in serum-free medium and seeded into the upper chamber of each transwell. This process was repeated for each of the groups. Before the transwells were filled, the lower chamber was filled with fetal bovine serum at a concentration of ten percent. In contrast to the migration experiment, the invasion assay was not pre-coated with Matrigel (BD Biosciences, Franklin Lakes, New Jersey, United States of America). Methanol (Serang Biotechnology, Chengdu, China) was used to fix the cells, and Crystal Violet (Amresco, Solon, Ohio, United States of America) was used to dye the cells. Both processes ended at the stated time. For the purpose of determining the number of cells, a microscope with a magnification of 200 × (Olympus Corporation, Tokyo, Japan) using five randomly selected fields was utilized.

### Western blot

A total of the proteins that were present in the cells and tissues were extracted, then electrophoretically separated using SDS-PAGE gel, and then transferred onto a PVDF membrane system. The membrane was incubated with specific primary antibodies against PPP2R2B, N‐cadherin, E‐cadherin, MMP‐9, β‐catenin, and GAPDH at a temperature of 4 degrees Celsius with shaking for 48 h. This was done after the membrane had been blocked with BSA at room temperature for two hours. The secondary antibodies were then added, and the mixture was left to incubate at room temperature for two hours. After that, the mixture was cleaned with TBST three times. Light-emitting solution was utilized for the exposure process. A reference for internal purposes was provided by GAPDH.

### In vivo* model*

The BALB/c mice, which were female, aged between four and six weeks, and weighed between eighteen and twenty grams, were housed in an environment that was free of pathogens. They were also given food and water that had been acidified on an ad libitum basis without any restrictions. A blinded randomization process was used to divide ten mice into two groups, each consisting of five mice, and then each group was given a separate therapy. Both statistical power and ethical requirements were taken into consideration while determining the size of the sample. An impartial examination was conducted in order to identify the criteria for the inclusion and removal of animals in compliance with the regulations that were established by the AAA-LAC. Subcutaneous injections of 2.0 × 10^7^ RT4 and RT4-ov-PPP2R2B cells administered in 100 μL of PBS were administered to the mice belonging to each group. All animal experiments were performed according to the Guidelines for the Care and Use of Laboratory Animals and were approved by the Institutional Animal Care and Use Committee of North China of Science and Technology Affiliated Hospital.

### Statistical analysis

R(version 4.0.2, R Core Team, Massachusetts, USA) and GraphPad Prism 6 (GraphPad Prism, San Diego, CA, USA) were utilized for each and every statistical analysis that was processed. Wilcoxon's test was utilized in order to make a comparison between the levels of MRG expression in normal tissues and those seen in BC. The Kruskal–Wallis test was utilized in order to investigate the degree of correlation that exists between MRGs and the clinical variables. An examination of the prognosis of MRGs was carried out with the use of the Kaplan–Meier survival analysis and the log rank test. For the purpose of determining the predictive value of MRGs in patients with BC, both univariate and multivariate Cox analyses were carried out. Statistical significance was established at a level of *p* < 0.05.

## Results

### Identification of the DE-MRGs between BC specimens and normal specimens and functional enrichment analysis

**Fig. 1:**
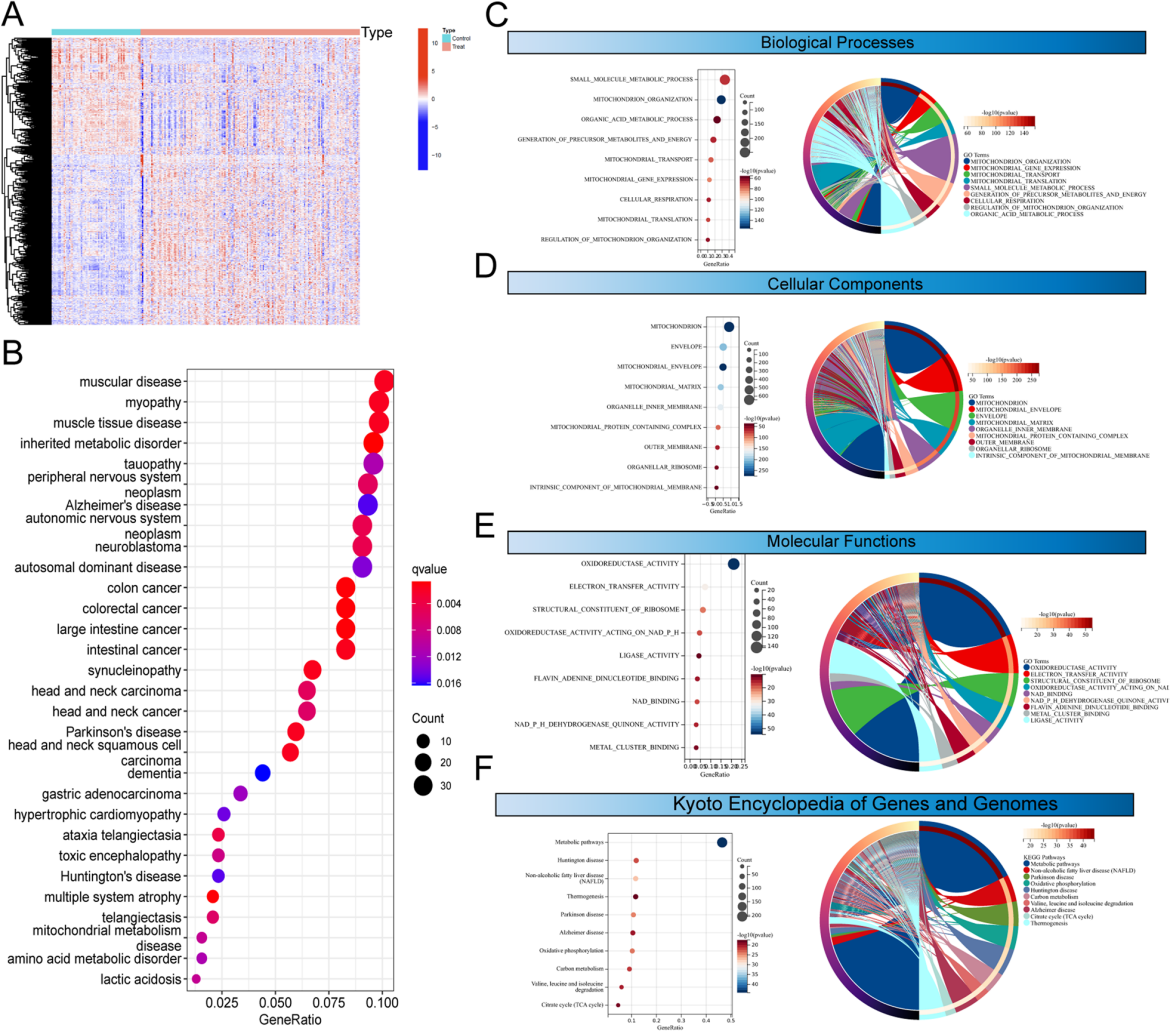
Identification and Functional Analysis of DE-MRGs in BC patients. **A** Through the application of the student’s t-test to GSE13507 datasets, a total of 752 DE-MRGs were discerned in BC specimens when compared to their normal counterparts. **B** DO analysis of DE-MRGs. **C**–**E** Gene Ontology (GO) analysis highlighted the functional roles of the 752 DE-MRGs. **F** KEGG pathway analysis revealed that the 752 DE-MRGs were significantly linked to various pathways

To screen functional genes involved in BC progression, we analyzed GSE13507 datasets using the student's t-test and identified 752 DE-MRGs in BC samples, including 313 down-regulated MRGs and 439 up-regulated MRGs (Fig. 1A). Then, we performed DO analysis to explore the possible association between 752 DE-MRGs and various diseases. As shown in Fig. 1B, we found that 752 DE-MRGs were mainly enriched in muscular disease, myopathy, muscle tissue disease, inherited metabolic disorder, tauopathy peripheral nervous system. In addition, the results of GO analysis revealed that t 752 DE-MRGs were mainly associated with Mitochondrion_Organization, Mitochondrial_Gene_Expression, Mitochondrial_Transport, Mitochondrion, Mitochondrial_Envelope, Envelope, Oxidoreductase_Activity, Electron_Transfer_Activity and Structural_Constituent_of_Ribosome (Fig. 1C–E). Moreover, based on the results of KEGG analysis, we found that 752 DE-MRGs were mainly related to Metabolic pathways, Huntington disease, Non-alcoholic fatty liver disease, Thermogenesis and Parkinson disease (Fig. 1F).

### Four DE-MRGs were identified as diagnostic genes for BC

**Fig. 2:**
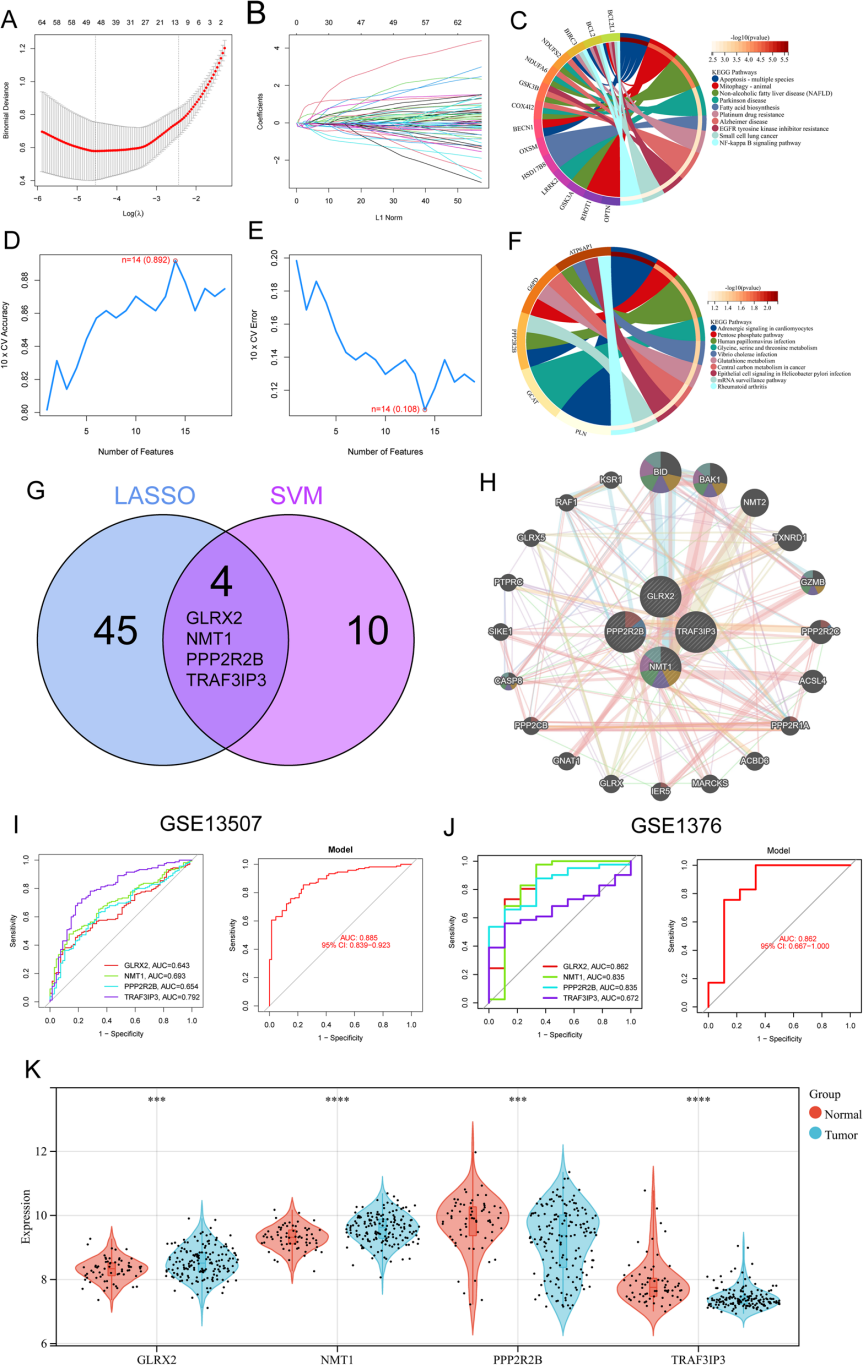
Identification and Validation of Diagnostic Genes for BC using Machine Learning Algorithms. **A**, **B** Utilizing the LASSO logistic regression algorithm with penalty parameter tuning via tenfold cross-validation, 49 BC-related features were selected from the DE-MRGs (**A**, **B**). **C** KEGG analysis of the 49 diagnostic genes. **D**, **E** The SVM-RFE algorithm identified an optimal combination of 14 feature genes to distinguish BC samples from normal samples. **F** KEGG analysis of the 14 genes. **G** Intersection of marker genes obtained from the LASSO and SVM-RFE models revealed four common genes (GLRX2, NMT1, PPP2R2B, and TRAF3IP3) for subsequent analysis. **H** The interacting network of GLRX2, NMT1, PPP2R2B, and TRAF3IP3 was constructed using the Genemania database, illustrating potential functional connections. **I** A logistic regression model incorporating the seven marker genes demonstrated robust performance, as indicated by ROC curves. **J** The diagnostic value of the model was validated in the GSE3167 dataset. **K** The expression patterns of GLRX2, NMT1, PPP2R2B, and TRAF3IP3 in BC samples and normal samples, based on GSE13507 datasets. ****p < 0.0001, ***p < 0.001, **p < 0.01, *p < 0.05. NS, not significant

We wanted to determine the diagnostic capability of DE-MRGs so that we could take into account the differences that exist between BC samples and normal samples respectively. In the following step, we identified the relevant DE-MRGs in the GSE13507 dataset by employing two distinct machine learning techniques, namely the LASSO and the SVM-RFE. These algorithms were used to differentiate between BC samples and normal samples. The selection of 49 BC-related features was accomplished through the utilization of the LASSO logistic regression technique, with penalty parameter adjustment being carried out through the process of tenfold cross-validation (Fig. 2A, B). KEGG analysis revealed that 49 diagnostic genes were mainly related to Apoptosis, Mitophagy, Non-alcoholic fatty liver disease, Parkinson disease and Fatty acid biosynthesis (Fig. 2C). Following that, we took the DE-MRGs and filtered them using the SVM-RFE algorithm and fourteen genes were determined to be the most suitable feature genes (Fig. 2D, E). In addition, the results of KEGG analysis revealed that 14 genes were mainly related to Adrenergic signaling in cardiomyocytes, Human papillomavirus infection, Pentose phosphate pathway, Glycine, serine and threonine metabolism and Vibrio cholerae infection (Fig. 2F). The marker genes that were acquired from the LASSO and SVM-RFE models were intersected, and as a result, four marker genes (GLRX2, NMT1, PPP2R2B, and TRAF3IP3) were identified for further investigation (Fig. 2G). By using Genemania database, the interacting network of GLRX2, NMT1, PPP2R2B and TRAF3IP3 was constructed and presented in Fig. 2H. Using the seven marker genes described above, we created a logistic regression model using the R package glm. The ROC assays demonstrated that the new model distinguished between normal and BC samples with AUC value of 0.885 (Fig. 2I). Moreover, the diagnostic value of the new model was further demonstrated in GSE3167 datasets (Fig. 2J). In addition, the expression pattern of GLRX2, NMT1, PPP2R2B and TRAF3IP3 in BC samples and normal samples based on GSE13507 datasets was shown in Fig. 2K.

### The expression pattern of GLRX2, NMT1, PPP2R2B and TRAF3IP3 in BC using TCGA datasets and their prognostic value

**Fig. 3:**
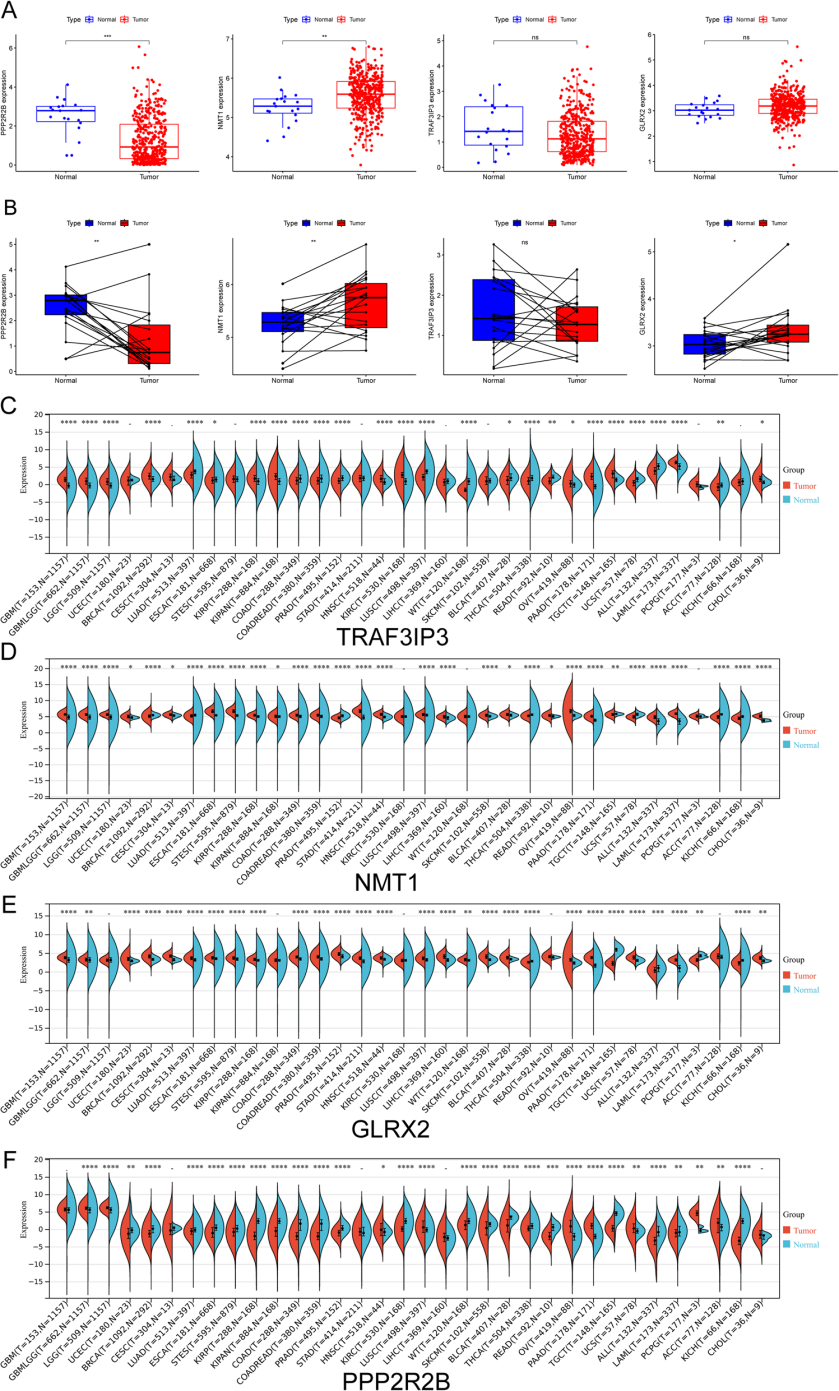
The expression pattern of GLRX2, NMT1, PPP2R2B and TRAF3IP3 in BC using TCGA datasets. **A**, **B** Dysregulated Expression of PPP2R2B and NMT1 in BC. **C**–**F** Pan-cancer analysis reveals dysregulation of GLRX2, NMT1, PPP2R2B, and TRAF3IP3 in various tumor types. ****p < 0.0001, ***p < 0.001, **p < 0.01, *p < 0.05

**Fig. 4:**
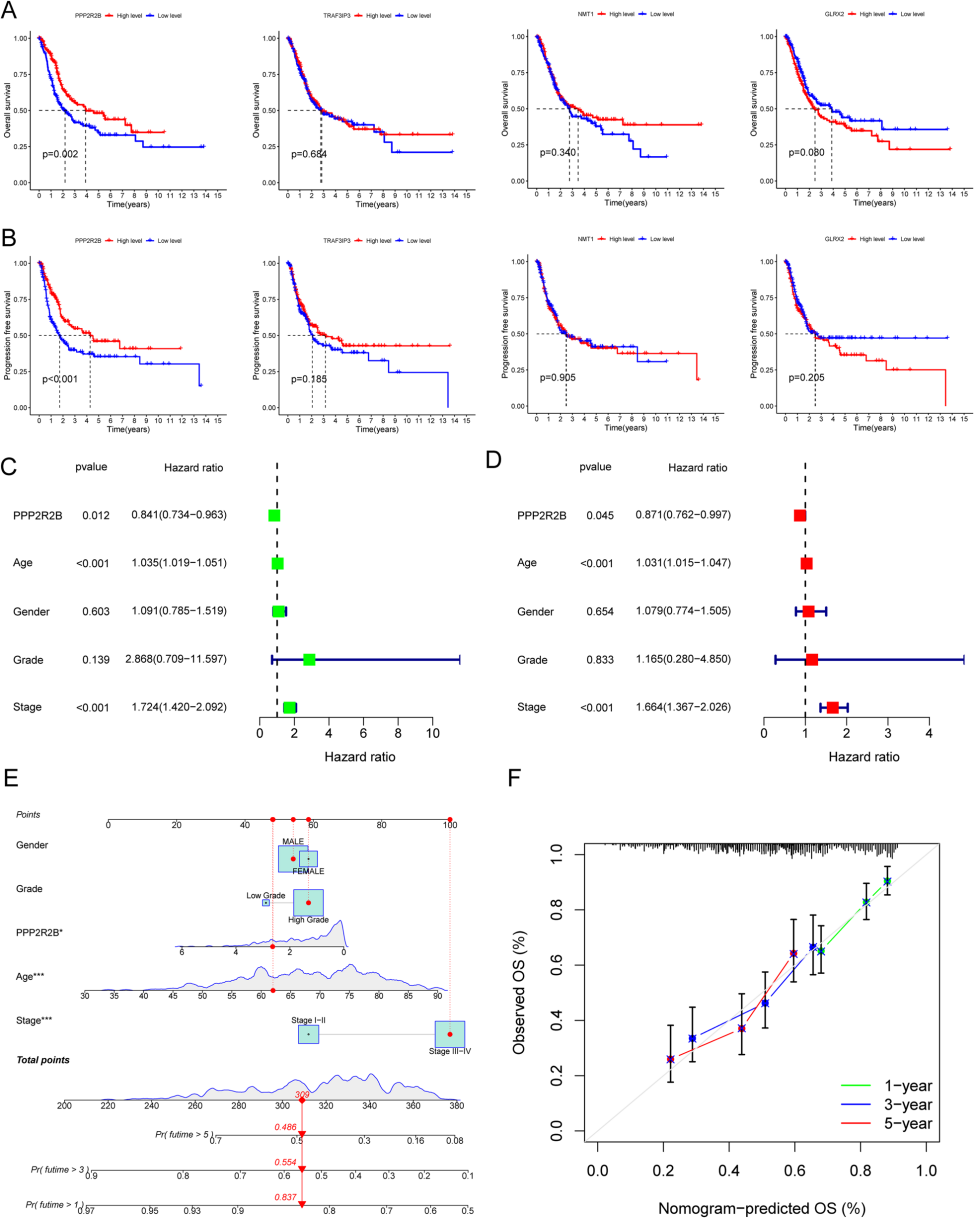
PPP2R2B as a Prognostic Biomarker in BC Patients. **A**, **B** Prognostic association of PPP2R2B with five-year overall survival and progression-free survival using Kaplan–Meier survival analysis. **C** Univariate Cox Proportional Hazard Analysis for PPP2R2B and other clinical factors. **D** Multivariate Cox Regression Analysis for PPP2R2B. **E** Construction of nomogram by the PPP2R2B expressions and clinical factors. **F** The calibration plot. ****p < 0.0001, ***p < 0.001, **p < 0.01, *p < 0.05

Furthermore, in order to investigate whether GLRX2, NMT1, PPP2R2B, and TRAF3IP3 displayed a dysregulated level in BC, we conducted further analyses on TCGA datasets. Our findings revealed that the expression of PPP2R2B and NMT1 displayed a dysregulated level when compared to normal samples (Fig. 3A, B). Based on the results of pan-cancer analysis, we found that GLRX2, NMT1, PPP2R2B and TRAF3IP3 exhibited a dysregulated level in many types of tumors, highlighting their potential roles in tumor progression (Fig. 3C–F). Then, we further explored the prognostic value of GLRX2, NMT1, PPP2R2B and TRAF3IP3 in BC patients. Importantly, we just observed that only PPP2R2B was associated with five year overall survival and progression free survival (Fig. 4A, B). After that, we concentrated our efforts on PPP2R2B. In order to further investigate the possibility of PPP2R2B being utilized as a novel biomarker for patients with BC, we carried out additional univariate and multivariate analyses using the proportional hazard model developed by Cox. According to the findings of the univariate analysis, the factors of age, stage, and PPP2R2B were substantially linked with the overall survival of patients with BC (Fig. 4C). Additionally, the multivariate Cox regression analysis demonstrated that the expression of PPP2R2B was an independent prognostic predictor for overall survival in patients (Fig. 4D). A quantitative tool that offers clinicians with the ability to predict the chance of overall survival at 1, 3, and 5 years for patients with BC is the expression level of PPP2R2B, which is an independent predictive risk factor (Fig. 4E, E).

### The association between PPP2R2B expression and clinical factors of BC patients

**Fig. 5:**
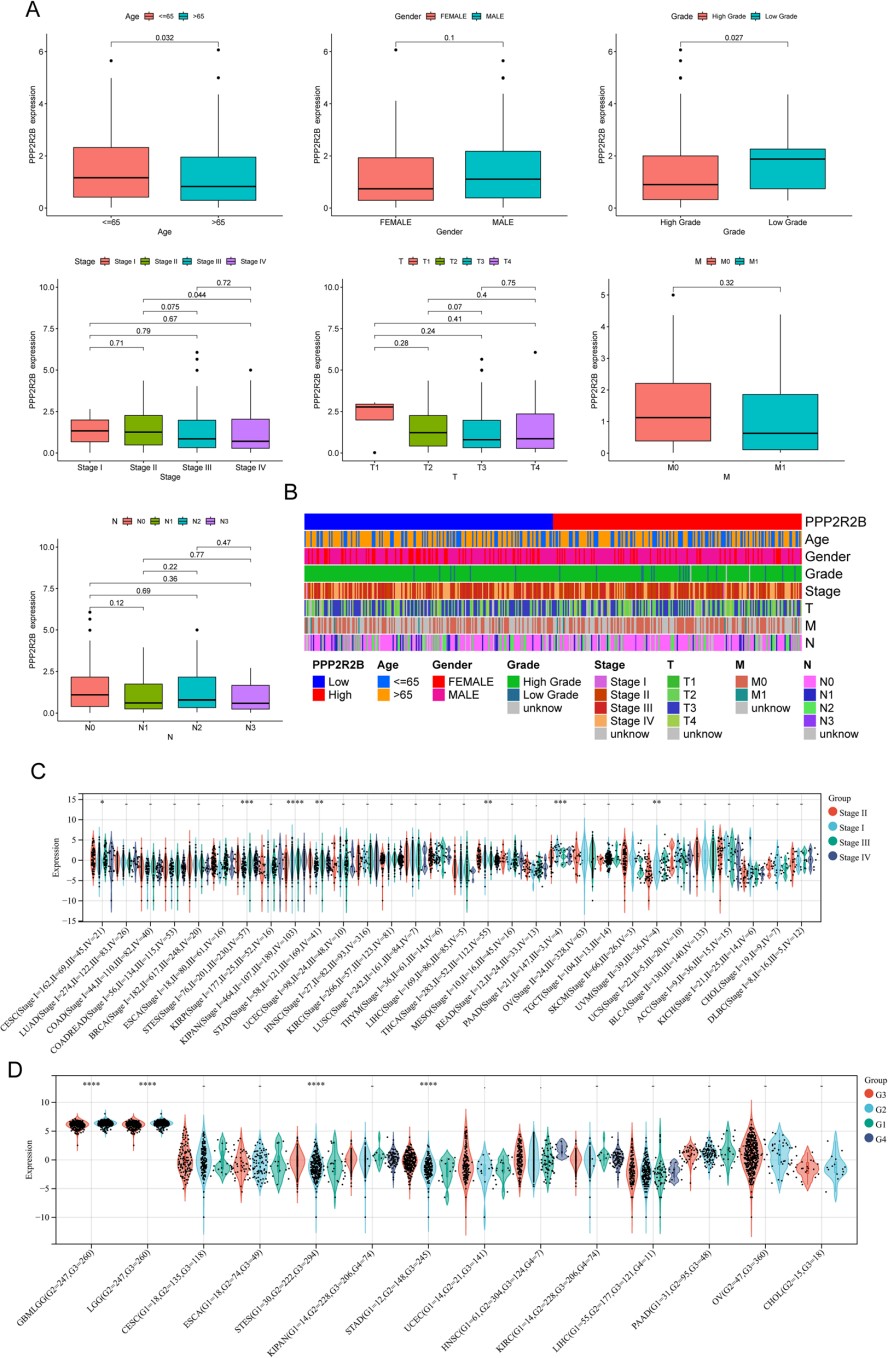
Association between PPP2R2B expression and clinical parameters in BC patients. **A** Scatter plot illustrating the relationship between PPP2R2B expression levels and age and grade in BC patients. Low PPP2R2B expression is associated with advanced age and higher tumor grade. **B** Heatmap Analysis of Clinical Characteristics in BC Patients Stratified by PPP2R2B Expression. Heatmap representation displaying the distribution of clinical characteristics in BC patients with high and low PPP2R2B expression levels. **C**, **D** Pancer analysis of PPP2R2B expression based on the clinical stage and grade. ****p < 0.0001, ***p < 0.001, **p < 0.01, *p < 0.05

Given the prognostic value of PPP2R2B in BC patients, we sought to elucidate the specific associations between PPP2R2B expression levels and clinical parameters in BC patients, with a focus on age, grade, clinical stage, T stage, M stage, and N stage. We found that low expression of PPP2R2B was associated with age and advanced grade. However, its expression was not clinical stage, T stage, M stage and N stage (Fig. 5A). Additionally, in order to illustrate the differences in the distribution of clinical variables across individuals whose PPP2R2B expression was high or low, we created a heatmap depicting the differences (Fig. 5B). In the pan-cancer analysis, PPP2R2B expression emerged as a potential indicator of clinical stages and grade in several tumor types. Notably, CESC, STES, KIPAD, GBM, and STAD exhibited varying degrees of association between PPP2R2B expression and the aforementioned clinical parameters (Fig. 5C, D). This broader perspective underscored the potential multifaceted role of PPP2R2B in tumorigenesis and suggests its relevance beyond BC. These results collectively contributed to our understanding of the intricate relationship between PPP2R2B expression and clinical factors in BC and other tumor types, paving the way for further exploration of PPP2R2B as a potential biomarker or therapeutic target.

### The biological functions of PPP2R2B in BC

**Fig. 6:**
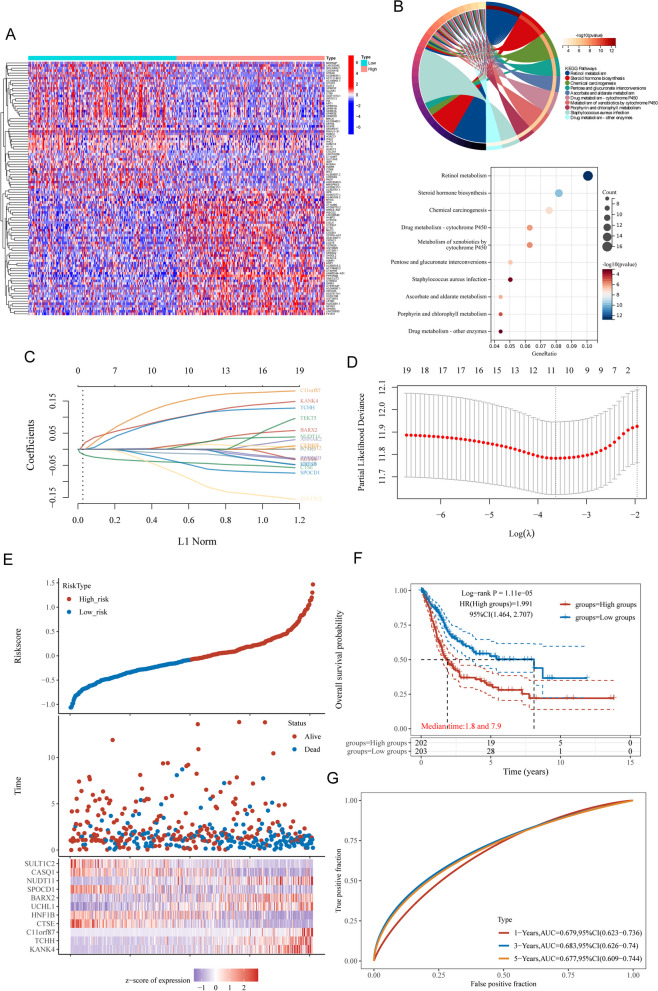
Construction of a prognostic signature based on DEGs between BC patients with low or high PPP2R2B expressions. **A** Differential Expression Analysis: A total of 761 DEGs were identified in BC patients with either high or low PPP2R2B expression levels. **B** KEGG Pathway Enrichment Analysis of 761 DEGs. **C** LASSO regression was performed, and the coefficients of the 11 selected DEGs were determined. **D** The model achieved the best fit when considering 11 out of the 19 DEGs. **E** The risk score formula was established based on the coefficients of the 11 selected DEGs. **F** The high-risk group, determined by the risk score model, exhibited significantly poorer overall survival compared to the low-risk group. **G** ROC analysis was conducted to evaluate the prognostic model's performance over time

After that, we isolated a variety of DEGs from patients whose PPP2R2B levels were either high or low. A total of 761 DEGs were screened (Fig. 6A). Then, we performed KEGG analysis and found that 761 DEGs were mainly enriched in Retinol metabolism, Chemical carcinogenesis and Steroid hormone biosynthesis (Fig. 6B).

### Construction of a prognostic signature based on DEGs between BC patients with low or high PPP2R2B expressions

We screened survival-related genes using DEGs between BC patients with low or high PPP2R2B expressions and confirmed 19 survival-related DEGs. Then, we thus carried out the LASSO regression to construct the prognostic signature in BC. The coefficient of 19 DEGs was presented in Fig. 6C. The model was able to obtain the best fit when 11 of the 19 DEGs were included (Fig. 6D). The formula used for risk score computation was as follows: Riskscore = (0.1099)*KANK4 + (0.1088)*TCHH + 0.1593)*C11orf87 + (-0.0429)*CTSE + (0.0142)*HNF1B + (0.0133)*UCHL1 + (0.0195)*BARX2 + (-0.0531)*SPOCD1 + (0.0181)*NUDT11 + (-9e-04)*CASQ1 + (-0.101)*SULT1C2. The risk score model used a median threshold to classify 405 BC tumor samples into low- or high-risk subgroups, and a heatmap of the 12 genes (12 DEGs) in BC was also created (Fig. 6E). Besides, the overall survival analysis presented that the high-risk group had notably poor overall survivals (Fig. 6F). Receiver operating characteristic (ROC) analysis that takes into account the passage of time was also used to evaluate the prognostic model's performance. One-year, three-year, and five-year overall survival (OS) area under the ROC curve (AUC) values were 0.679, 0.683, and 0.677, respectively (Fig. 6G).

### PPP2R2B was lowly expressed in BC specimens and its overexpression suppressed the proliferation and metastasis of BC cells

**Fig. 7:**
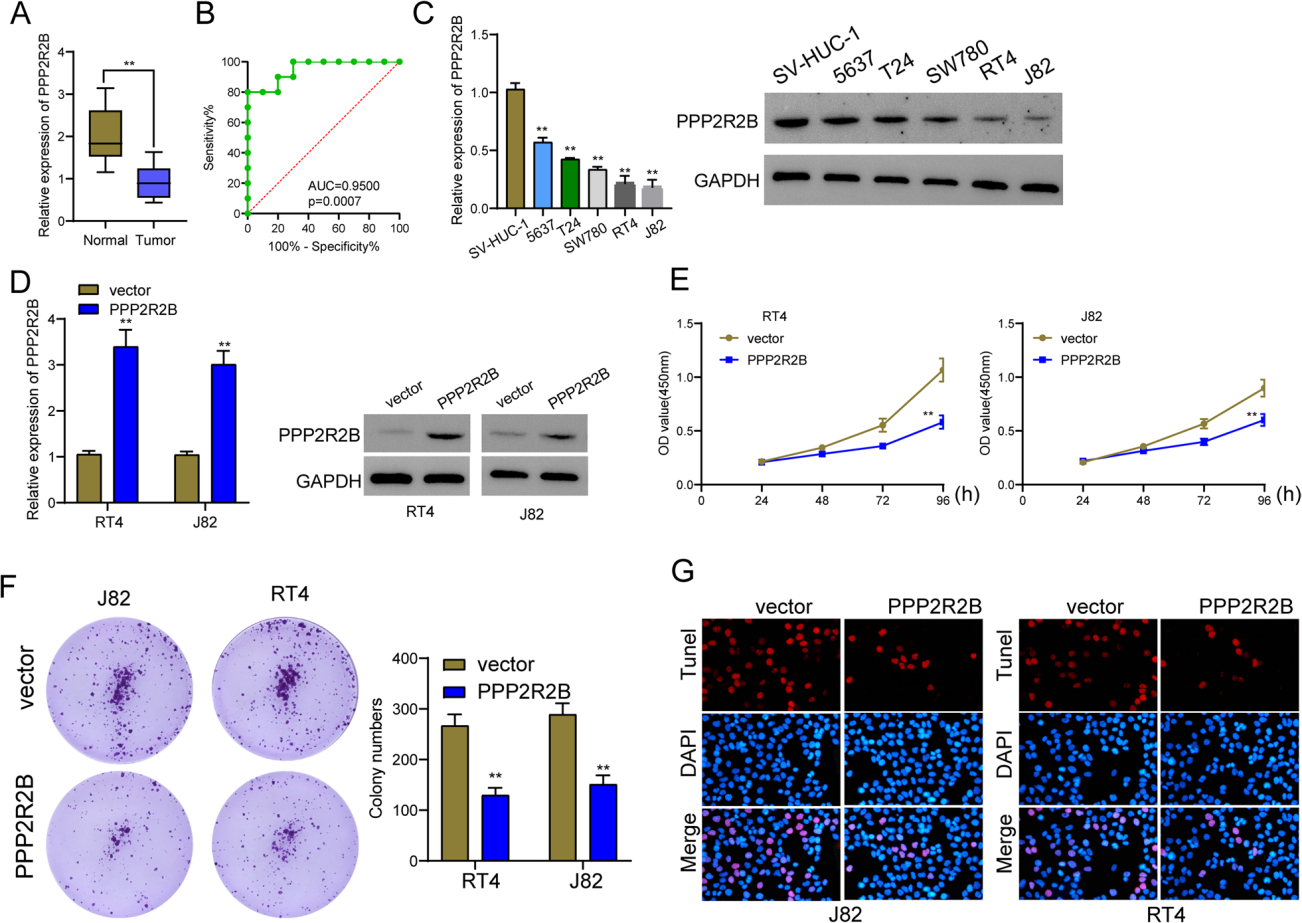
PPP2R2B was low expressed in BC and its overexpression suppressed the proliferation of BC cells. **A** The expression of PPP2R2B in 10 pairs of BC and normal specimens using RT-PCR. **B** ROC analysis determining the diagnostic value of PPP2R2B expression in BC patients. **C** RT-PCR and western blot for the levels of PPP2R2B in five BC cell lines and normal cells. **D** The expression of PPP2R2B was distinctly increased in RT4 and J82 cells after the transfection of PPP2R2B plasmid. **E** RT4 and J82 cells that had been transfected with the PPP2R2B plasmid or vector were subjected to CCK-8 assay. **F** colony formation assays of RT4 and J82 cells. **G** Apoptosis was identified through the utilization of the TUNEL test. ***p < 0.001, **p < 0.01, *p < 0.05

**Fig. 8:**
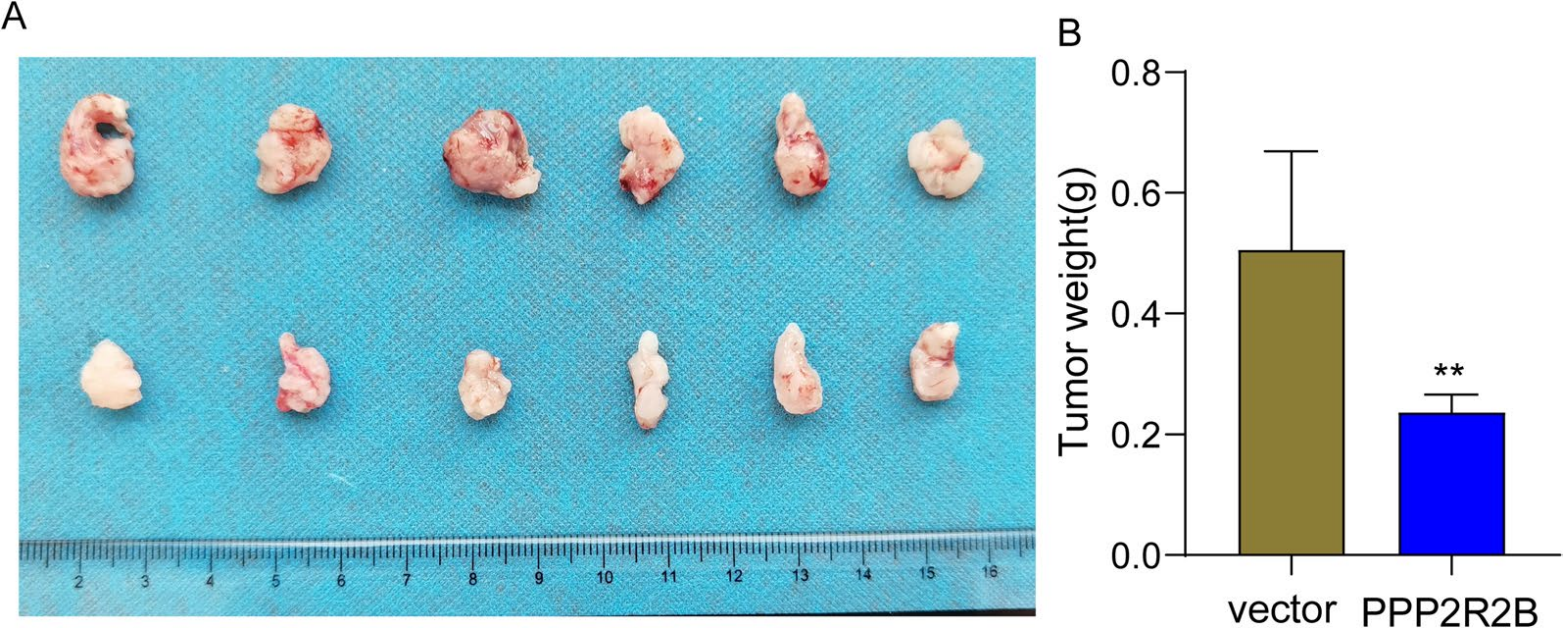
PPP2R2B overexpression inhibited tumor growth in vivo. **A** The image of xenografts isolated from mice one month. **B** The tumor weights. **p < 0.01

**Fig. 9:**
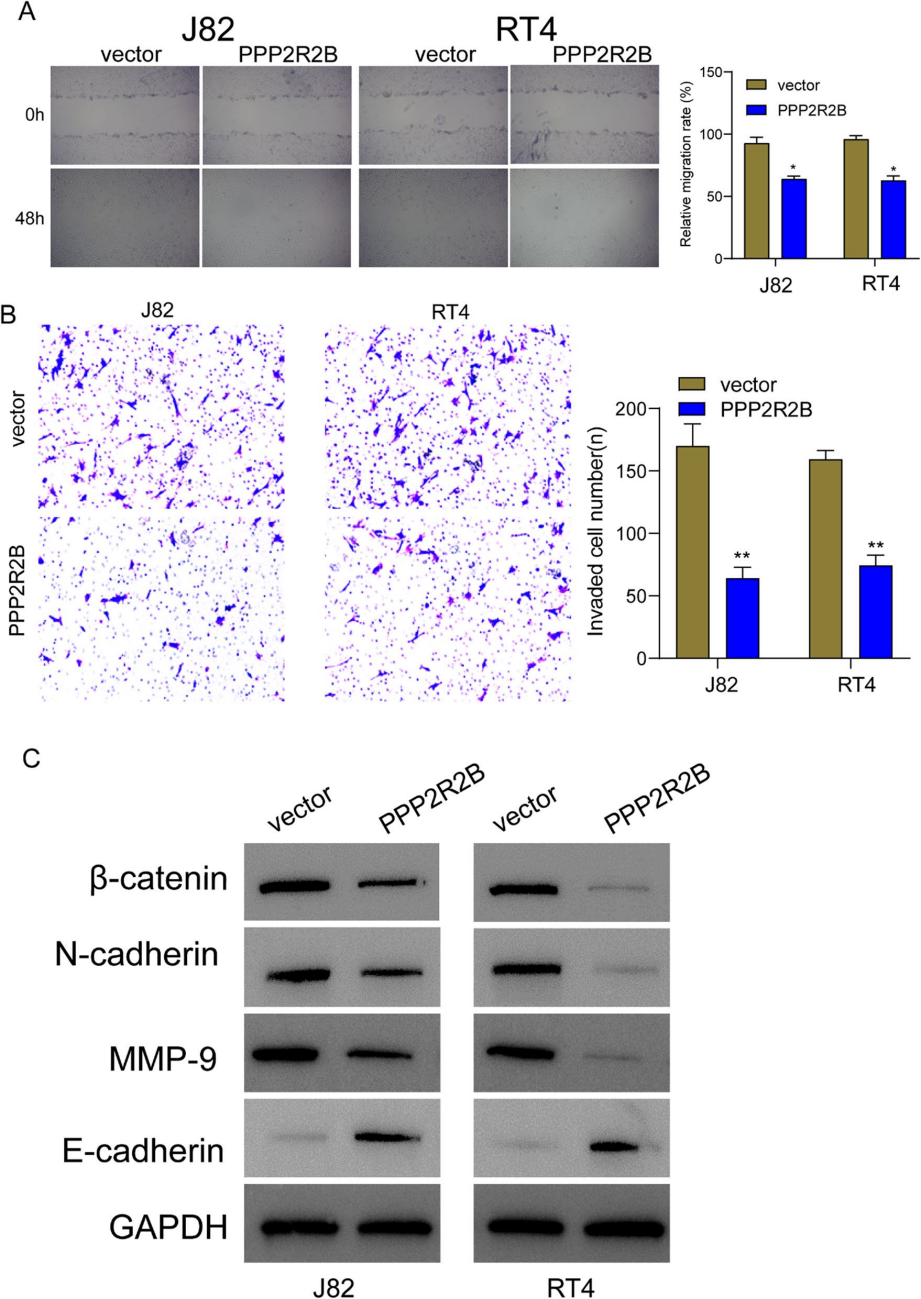
Overexpression of PPP2R2B suppressed migration and invasion of BC cells via Wnt/β‐catenin/EMT pathway. **A** The evaluation of cell migration of RT4 and J82 was carried out using wound healing tests following the overexpression of PPP2R2B. **B** In order to investigate the process of cell invasion in RT4 and J82 cells, transwell invasion experiments were utilized. **C** Following the overexpression of PPP2R2B, the levels of N‐cadherin, E‐cadherin, MMP‐9, and β‐catenin in RT4 and J82 were determined through the utilization of Western blot detection. **p < 0.01, *p < 0.05

Based on the above results from TCGA datasets, we reported PPP2R2B expression was down-regulated in BC patients. Then, we performed RT-PCR using our cohort and the results further confirmed that the expression of PPP2R2B was distinctly decreased in BC specimens compared with non-tumor specimens (Fig. 7A). The diagnostic value of PPP2R2B in screening BC specimens from normal specimens was also confirmed using ROC assays with AUC = 0.950 (Fig. 7B). Then, we also performed RT-PCR and western blot to examine its levels in five BC cell lines. As shown in Fig. 7C, we observed that the expressions of PPP2R2B at both mRNA and protein levels were distinctly decreased in five BC cell lines compared with SV-HUC-1. Then, we constructed the PPP2R2B overexpression plasmid and the results of RT-PCR confirmed that it distinctly increased the expression of PPP2R2B in RT4 and J82 cells (Fig. 7D). The findings of the CCK-8 tests and the clonogenic assays indicated that the overexpression of PPP2R2B significantly inhibited the proliferation of RT4 and J82 cells (Fig. 7E–F). Moreover, according to the results of TUNEL assays, the overexpression of PPP2R2B led to a considerable increase in the amount of cell death in RT4 and J82 cells (Fig. 7G). Moreover, we performed in vivo experiments. The weights of the tumors present in the group that had PPP2R2B overexpression were noticeably lower than those found in the group that had vector (Fig. 8A, B). In addition, we further explored whether PPP2R2B may exhibit a functional role in the metastasis of BC cells. PPP2R2B overexpression was found to have a significant suppressive effect on the migration and invasion of RT4 and J82 cells, as demonstrated by the findings of the wound healing experiment and the transwell assays (Fig. 9A, B).

### PPP2R2B overexpression suppressed the activity of Wnt/β‐catenin/EMT pathway

There are numerous biological processes that require the Wnt/β‐catenin/EMT system, which is an essential cellular signaling route [26, 27]. These activities include embryonic development, tissue regeneration, cell proliferation, and migration. It is essential for the invasion and spread of malignancies that the EMT process is carried out. The activation of the Wnt/β‐catenin pathway can induce EMT, causing tumor cells to migrate from their original location and acquire the ability to invade other tissues [28, 29]. By altering the morphology and function of cells, EMT facilitates the tumor cells' easier traversal through vascular walls, allowing them to enter the bloodstream or lymphatic system, thus achieving distant organ metastasis. To further explore the potential mechanisms involved in the anti-oncogenic roles of PPP2R2B in BC progression, we performed western blot and found that overexpression of PPP2R2B suppressed the levels of MMP‐9, β‐catenin and N‐cadherin, while promoting the expression of E‐cadherin (Fig. 9C).

## Discussion

BC is a common malignancy of the urinary system, and early diagnosis is crucial for effective treatment and patient prognosis [30]. The primary clinical diagnostic markers currently investigated include NMP22, UroVysion FISH testing, and some biomarkers assisted by cystoscopy [31, 32]. NMP22, a nuclear matrix protein, may be elevated in urine and associated with BC. Despite its simplicity and non-invasiveness, NMP22 exhibits lower specificity, posing a risk of false-positive results [33]. UroVysion FISH testing diagnoses BC by detecting chromosomal abnormalities in urine, demonstrating a certain level of accuracy in early diagnosis and monitoring high-risk patients [34]. However, its widespread application is limited by its high cost and the need for specialized technical expertise. On the flip side, certain studies concentrate on cystoscopy-assisted biomarkers like BLCA-4 and UBC, aiming to augment the detection rate of BC when used in conjunction with cystoscopy. However, it is important to note that the sensitivity and specificity of these markers still necessitate additional validation. There is an ongoing need for the further demonstration of more sensitive biomarkers for BC.

A significant correlation exists between BC and mitochondria, particularly in the realms of cellular energy metabolism, oxidative stress, and the regulation of apoptosis. In BC, cells exhibit a heightened demand for energy, and mitochondria, serving as the cellular energy center, generate adenosine triphosphate (ATP) through the process of oxidative phosphorylation to meet the rapid proliferation and growth requirements [35, 36]. However, oxidative stress may lead to mitochondrial dysfunction, causing damage to mitochondrial DNA and consequently impacting normal cellular metabolism. Additionally, mitochondria play a crucial role in apoptosis regulation, where the release of signals under normal circumstances guides cell death [37, 38]. Yet, certain BC cells may evade the normal apoptotic mechanisms by modulating mitochondrial apoptotic pathways, promoting the survival and dissemination of cancer cells. Studies have also identified gene variations related to mitochondrial DNA in BC patients, potentially influencing the structure and function of mitochondria and, consequently, impacting the progression of BC [39–41]. In the analysis of GSE13507 datasets, we identified 752 dysregulated DE-MRGs in BC, with 313 down-regulated and 439 up-regulated genes. These genes were associated with various diseases, including muscular disease and myopathy, and were functionally linked to mitochondrial organization, gene expression, and metabolic pathways, as revealed by enrichment analyses. Then, we performed LASSO and SVM-RFE, and identified four critical diagnostic genes for BC, including GLRX2, NMT1, PPP2R2B and TRAF3IP3. Then, we developed a diagnostic model using GLRX2, NMT1, PPP2R2B and TRAF3IP3, which was confirmed to exhibit a strong ability in screening BC specimens from normal specimens. Our findings underscored the potential of these genes as valuable biomarkers for effective BC screening.

To further identify the critical gene involved in BC progression, we performed survival analysis using GLRX2, NMT1, PPP2R2B and TRAF3IP3 based on TCGA datasets, and only low PPP2R2B expression was associated with shorter OS and PFS, suggesting it as an anti-oncogene in BC patients. Protein Phosphatase 2A (PP2A) is a crucial protein phosphatase belonging to the Protein Phosphatase 2 (PPP) family [42, 43]. PP2A plays a vital role in regulating various biological processes within the cell, including the cell cycle, apoptosis, cell signaling pathways, and cell differentiation. It represents the primary serine/threonine phosphatase in the cell and accounts for a significant portion of cellular phosphatase activity [44, 45]. PP2A is actively involved in the control of diverse cellular processes, including cell cycle progression, apoptosis, cellular signal transduction pathways (such as Wnt, MAPK, etc.), and cell differentiation. It stands out as a key regulator in these fundamental cellular activities. The aberrant expression or dysfunction of PP2A has been linked to the initiation of various diseases, including several types of cancer. This emphasizes the crucial role of PP2A in preserving cellular homeostasis and highlights its potential as a target for therapeutic interventions in conditions marked by disrupted cellular processes. The protein encoded by PPP2R2B is a member of the B subunit family of Protein Phosphatase 2A (PP2A). The B subunit is a structural component of the PP2A complex [46–48]. The PP2A complex consists of a catalytic subunit (C subunit), a structural subunit (A subunit), and one or more regulatory subunits (B subunits). The presence of the B subunit allows PP2A to interact with different substrates, enabling it to perform various functions in different cellular processes. Previously, several studies have reported that the dysregulation of PPP2R2B was involved in the progression of several tumors [49, 50]. For instance, Li et al. reported that when compared to normal breast tissues, the expression of PPP2R2B was shown to be considerably downregulated, and it was associated with a bad prognosis. Moreover, its overexpression was distinctly associated with immune evasion [51]. However, the expression and function of PPP2R2B in BC have not been reported. Our significant findings contribute substantial confirmation to the notion that PPP2R2B expression stands as an independent predictor for overall survival in individuals diagnosed with BC. Moreover, the Nomogram analysis unveiled that the expression level of PPP2R2B, serving as an independent predictive risk factor, provides a quantitative tool for healthcare professionals to assess the likelihood of progression-free survival at 1, 3, and 5 years for BC patients. This study illuminates the potential of PPP2R2B expression as a novel and valuable predictive biomarker for individuals grappling with bladder cancer, offering clinicians a robust tool for prognostic evaluation and personalized treatment strategies.

Based on the above findings, we confirmed PPP2R2B as an anti-oncogene in BC progression. Then, we further performed RT-PCR using our cohort and confirmed that PPP2R2B expression was distinctly decreased in both BC specimens and cell lines. In addition, ROC assays also confirmed its diagnostic value in BC patients. Then, we performed functional experiments and observed that overexpression of PPP2R2B distinctly suppressed the proliferation, migration and invasion of BC cells, while promoted the apoptosis, further confirming its anti-oncogene roles in BC progression. Wnt/β-catenin is a signaling system that plays a vital role in numerous biological functions, such as the regulation of cell proliferation and differentiation, as well as embryonic development and tissue homeostasis [29, 52]. Wnt, which is a family of glycoproteins that are secreted, and β-catenin, which is a protein that plays a vital role in the transmission of Wnt signals, are the two main components; the route takes its name from these two components [53]. In situations where Wnt signaling is not present, β-catenin is typically found in the cytoplasm, where it forms a complex with a destruction complex. Axin, APC (adenomatous polyposis coli), GSK-3β (glycogen synthase kinase-3β), and CK1 (casein kinase 1) are some of the proteins that are included in this destruction complex [54, 55]. This complex serves as a marker for the breakdown of β-catenin. In addition, the activation of the Wnt/β-catenin pathway is linked to the process of EMT. There is a change in the cellular state known as EMT, which results in epithelial cells losing their polarity and acquiring features that are typical of mesenchymal cells. This transformation endows cells with enhanced migration and invasive capabilities, contributing to the spread and metastasis of tumors. In certain cancers, the activation of the Wnt/β-catenin pathway is closely linked to the occurrence of EMT, collectively participating in the invasive and metastatic processes of tumors [26, 56, 57]. In this study, we found that overexpression of PPP2R2B suppressed the expressions of N‐cadherin, β‐catenin and MMP‐9 and while promoting the expression of E‐cadherin, suggesting PPP2R2B overexpression promoted BC progression via suppressing Wnt/β‐catenin/EMT pathway. By investigating how PPP2R2B influences the Wnt/β-catenin/EMT pathway, we can gain a deeper understanding of the role of this pathway in the development of BC. Our findings contributed to unraveling the complexity of tumor biology and provides a direction for future research.

## Conclusion

We identified a new BC-related mitochondria-related gene PPP2R2B which was lowly expressed in BC. In addition, we firstly reported the diagnostic and prognostic value of PPP2R2B in BC patients. Functionally, we confirmed that PPP2R2B overexpression distinctly suppressed the proliferation and metastasis of BC cells via Wnt/β-catenin/EMT pathway. In summary, these findings offer new insights into the biological characteristics of BC, highlighting PPP2R2B as a crucial regulatory factor. Simultaneously, this study provides important clues and directions for future investigations into the molecular mechanisms of BC, the development of novel therapeutic strategies, and the enhancement of patient prognosis assessment.

## Data Availability

The datasets generated during and/or analyzed during the current study are available from the corresponding author upon reasonable request.

## References

[1] Miller KD, Nogueira L, Devasia T, Mariotto AB, Yabroff KR, Jemal A, Kramer J, Siegel RL. Cancer treatment and survivorship statistics, 2022. CA 2022;72(5):409–436. 10.3322/caac.21731 35736631

[2] Xia C, Dong X, Li H, Cao M, Sun D, He S, Yang F, Yan X, Zhang S, Li N (2022). Cancer statistics in China and United States, 2022: profiles, trends, and determinants. Chin Med J.

[3] Compérat E, Amin MB, Cathomas R, Choudhury A, De Santis M, Kamat A, Stenzl A, Thoeny HC, Witjes JA (2022). Current best practice for bladder cancer: a narrative review of diagnostics and treatments. Lancet (London, England).

[4] Martinez Rodriguez RH, Buisan Rueda O, Ibarz L (2017). Bladder cancer: present and future. Med Clin.

[5] Dobruch J, Oszczudłowski M: Bladder cancer: current challenges and future directions. Medicina (Kaunas, Lithuania) 2021;57(8). 10.3390/medicina57080749 PMC8402079 34440955

[6] Kobayashi T, Owczarek TB, McKiernan JM, Abate-Shen C (2015). Modelling bladder cancer in mice: opportunities and challenges. Nat Rev Cancer.

[7] Greener JG, Kandathil SM, Moffat L, Jones DT (2022). A guide to machine learning for biologists. Nat Rev Mol Cell Biol.

[8] Mai H, Le TC, Chen D, Winkler DA, Caruso RA (2022). Machine learning for electrocatalyst and photocatalyst design and discovery. Chem Rev.

[9] Myszczynska MA, Ojamies PN, Lacoste AMB, Neil D, Saffari A, Mead R, Hautbergue GM, Holbrook JD, Ferraiuolo L (2020). Applications of machine learning to diagnosis and treatment of neurodegenerative diseases. Nat Rev Neurol.

[10] Zou J, Huss M, Abid A, Mohammadi P, Torkamani A, Telenti A (2019). A primer on deep learning in genomics. Nat Genet.

[11] Theodosiou AA, Read RC (2023). Artificial intelligence, machine learning and deep learning: potential resources for the infection clinician. J Infect.

[12] Al Amir Dache Z, Thierry AR. Mitochondria-derived cell-to-cell communication. Cell Rep 2023;42(7):112728. 10.1016/j.celrep.2023.112728 37440408

[13] Nunnari J, Suomalainen A (2012). Mitochondria: in sickness and in health. Cell.

[14] Duan C, Liu R, Kuang L, Zhang Z, Hou D, Zheng D, Xiang X, Huang H, Liu L, Li T. Activated Drp1 initiates the formation of endoplasmic reticulum-mitochondrial contacts via Shrm4-mediated actin bundling. Adv Sci (Weinheim, Baden-Wurttemberg, Germany) 2023;10(36):e2304885. 10.1002/advs.202304885 PMC10754141 37909346

[15] Ng MYW, Wai T, Simonsen A (2021). Quality control of the mitochondrion. Dev Cell.

[16] Averbeck D, Rodriguez-Lafrasse C. Role of mitochondria in radiation responses: epigenetic, metabolic, and signaling impacts. Int J Mol Sci 2021;22(20). 10.3390/ijms222011047 PMC8541263 34681703

[17] Oyewole AO, Birch-Machin MA (2015). Mitochondria-targeted antioxidants. FASEB J.

[18] Zeng X, Zhang YD, Ma RY, Chen YJ, Xiang XM, Hou DY, Li XH, Huang H, Li T, Duan CY (2022). Activated Drp1 regulates p62-mediated autophagic flux and aggravates inflammation in cerebral ischemia-reperfusion via the ROS-RIP1/RIP3-exosome axis. Mil Med Res.

[19] Liu Y, Chen C, Wang X, Sun Y, Zhang J, Chen J, Shi Y. An epigenetic role of mitochondria in cancer. Cells 2022;11(16). 10.3390/cells11162518 PMC9406960 36010594

[20] Porporato PE, Filigheddu N, Pedro JMB, Kroemer G, Galluzzi L (2018). Mitochondrial metabolism and cancer. Cell Res.

[21] Borcherding N, Brestoff JR (2023). The power and potential of mitochondria transfer. Nature.

[22] Zong WX, Rabinowitz JD, White E (2016). Mitochondria and cancer. Mol Cell.

[23] Yu G, Wang LG, Han Y, He QY (2012). clusterProfiler: an R package for comparing biological themes among gene clusters. OMICS.

[24] Sanz H, Valim C, Vegas E, Oller JM, Reverter F (2018). SVM-RFE: selection and visualization of the most relevant features through non-linear kernels. BMC Bioinf.

[25] Cheng C, Hua ZC (2020). Lasso peptides: heterologous production and potential medical application. Front Bioeng Biotechnol.

[26] Gonzalez DM, Medici D. Signaling mechanisms of the epithelial-mesenchymal transition. Sci Signal 2014;7(344):re8. 10.1126/scisignal.2005189 PMC4372086 25249658

[27] Ang HL, Mohan CD, Shanmugam MK, Leong HC, Makvandi P, Rangappa KS, Bishayee A, Kumar AP, Sethi G (2023). Mechanism of epithelial-mesenchymal transition in cancer and its regulation by natural compounds. Med Res Rev.

[28] Hayat R, Manzoor M, Hussain A (2022). Wnt signaling pathway: a comprehensive review. Cell Biol Int.

[29] Nusse R, Clevers H (2017). Wnt/β-Catenin signaling, disease, and emerging therapeutic modalities. Cell.

[30] Lenis AT, Lec PM, Chamie K, Mshs MD (2020). Bladder cancer: a review. JAMA.

[31] Dyrskjøt L, Hansel DE, Efstathiou JA, Knowles MA, Galsky MD, Teoh J, Theodorescu D (2023). Bladder cancer. Nat Rev Dis Prim.

[32] Varchulová Nováková Z, Kuniaková M, Žiaran S, Harsányi Š (2023). Molecular biomarkers of bladder cancer: a mini-review. Physiol Res.

[33] Soorojebally Y, Neuzillet Y, Roumiguié M, Lamy PJ, Allory Y, Descotes F, Ferlicot S, Kassab-Chahmi D, Oudard S, Rébillard X (2023). Urinary biomarkers for bladder cancer diagnosis and NMIBC follow-up: a systematic review. World J Urol.

[34] Halling KC, Kipp BR (2008). Bladder cancer detection using FISH (UroVysion assay). Adv Anat Pathol.

[35] Woolbright BL, Ayres M, Taylor JA (2018). Metabolic changes in bladder cancer. Urol Oncol.

[36] Dong X, Zeng Y, Zhang Z, Fu J, You L, He Y, Hao Y, Gu Z, Yu Z, Qu C (2021). Hypericin-mediated photodynamic therapy for the treatment of cancer: a review. J Pharm Pharmacol.

[37] Pop-Bica C, Gulei D, Cojocneanu-Petric R, Braicu C, Petrut B, Berindan-Neagoe I. Understanding the role of non-coding RNAs in bladder cancer: from dark matter to valuable therapeutic targets. Int J Mol Sci 2017;18(7). 10.3390/ijms18071514 PMC5536004 28703782

[38] Yang Y, Karakhanova S, Hartwig W, D'Haese JG, Philippov PP, Werner J, Bazhin AV (2016). Mitochondria and mitochondrial ROS in cancer: novel targets for anticancer therapy. J Cell Physiol.

[39] Poole LP, Macleod KF (2021). Mitophagy in tumorigenesis and metastasis. Cell Mol Life Sci CMLS.

[40] Sainero-Alcolado L, Liaño-Pons J, Ruiz-Pérez MV, Arsenian-Henriksson M (2022). Targeting mitochondrial metabolism for precision medicine in cancer. Cell Death Differ.

[41] Luo Y, Ma J, Lu W. The significance of mitochondrial dysfunction in cancer. Int J Mol Sci 2020;21(16). 10.3390/ijms21165598 PMC7460667 32764295

[42] Shao L, Ma Y, Fang Q, Huang Z, Wan S, Wang J, Yang L (2021). Role of protein phosphatase 2A in kidney disease (Review). Exp Ther Med.

[43] O'Connor CM, Perl A, Leonard D, Sangodkar J, Narla G (2018). Therapeutic targeting of PP2A. Int J Biochem Cell Biol.

[44] Dai S, Wang C, Zhao X, Ma C, Fu K, Liu Y, Peng C, Li Y (2023). Cucurbitacin B: a review of its pharmacology, toxicity, and pharmacokinetics. Pharmacol Res.

[45] Ronk H, Rosenblum JS, Kung T, Zhuang Z (2022). Targeting PP2A for cancer therapeutic modulation. Cancer Biol Med.

[46] Sontag JM, Sontag E (2014). Protein phosphatase 2A dysfunction in Alzheimer's disease. Front Mol Neurosci.

[47] Roy S, Batra L (2023). Protein phosphatase 2A: role in T cells and diseases. J Immunol Res.

[48] Clark AR, Ohlmeyer M (2019). Protein phosphatase 2A as a therapeutic target in inflammation and neurodegeneration. Pharmacol Ther.

[49] Malvi P, Chava S, Cai G, Hu K, Zhu LJ, Edwards YJK, Green MR, Gupta R, Wajapeyee N (2023). HOXC6 drives a therapeutically targetable pancreatic cancer growth and metastasis pathway by regulating MSK1 and PPP2R2B. Cell Rep Med.

[50] Vazquez A, Kulkarni D, Grochola LF, Bond GL, Barnard N, Toppmeyer D, Levine AJ, Hirshfield KM (2011). A genetic variant in a PP2A regulatory subunit encoded by the PPP2R2B gene associates with altered breast cancer risk and recurrence. Int J Cancer.

[51] Li Z, Li Y, Wang X, Yang Q (2021). PPP2R2B downregulation is associated with immune evasion and predicts poor clinical outcomes in triple-negative breast cancer. Cancer Cell Int.

[52] Zhang Y, Wang X (2020). Targeting the Wnt/β-catenin signaling pathway in cancer. J Hematol Oncol.

[53] Yu F, Yu C, Li F, Zuo Y, Wang Y, Yao L, Wu C, Wang C, Ye L (2021). Wnt/β-catenin signaling in cancers and targeted therapies. Signal Transduct Target Ther.

[54] Clevers H, Nusse R (2012). Wnt/β-catenin signaling and disease. Cell.

[55] Perugorria MJ, Olaizola P, Labiano I, Esparza-Baquer A, Marzioni M, Marin JJG, Bujanda L, Banales JM (2019). Wnt-β-catenin signalling in liver development, health and disease. Nat Rev Gastroenterol Hepatol.

[56] Zhao H, Ming T, Tang S, Ren S, Yang H, Liu M, Tao Q, Xu H (2022). Wnt signaling in colorectal cancer: pathogenic role and therapeutic target. Mol Cancer.

[57] Klauzinska M, Castro NP, Rangel MC, Spike BT, Gray PC, Bertolette D, Cuttitta F, Salomon D (2014). The multifaceted role of the embryonic gene Cripto-1 in cancer, stem cells and epithelial-mesenchymal transition. Semin Cancer Biol.

