# Correction: Comprehensive analysis of mitochondria-related genes indicates that PPP2R2B is a novel biomarker and promotes the progression of bladder cancer via Wnt signaling pathway

**DOI:** 10.1186/s13062-024-00474-1

**Published:** 2024-04-18

**Authors:** Du Shen, Shaosan Kang

**Affiliations:** 1 College of Clinic Medical, North China University of Science and Technology, Tangshan, China; 2 North China of Science and Technology Affiliated Hospital, Tangshan, China

**Correction: Shen and Kang Biology Direct (2024) 19:17** 10.1186/s13062-024-00461-6

After publication of this article [1], it was brought to our attention that the Figure 9 need to be corrected.

The Incorrect Figure 9 is:
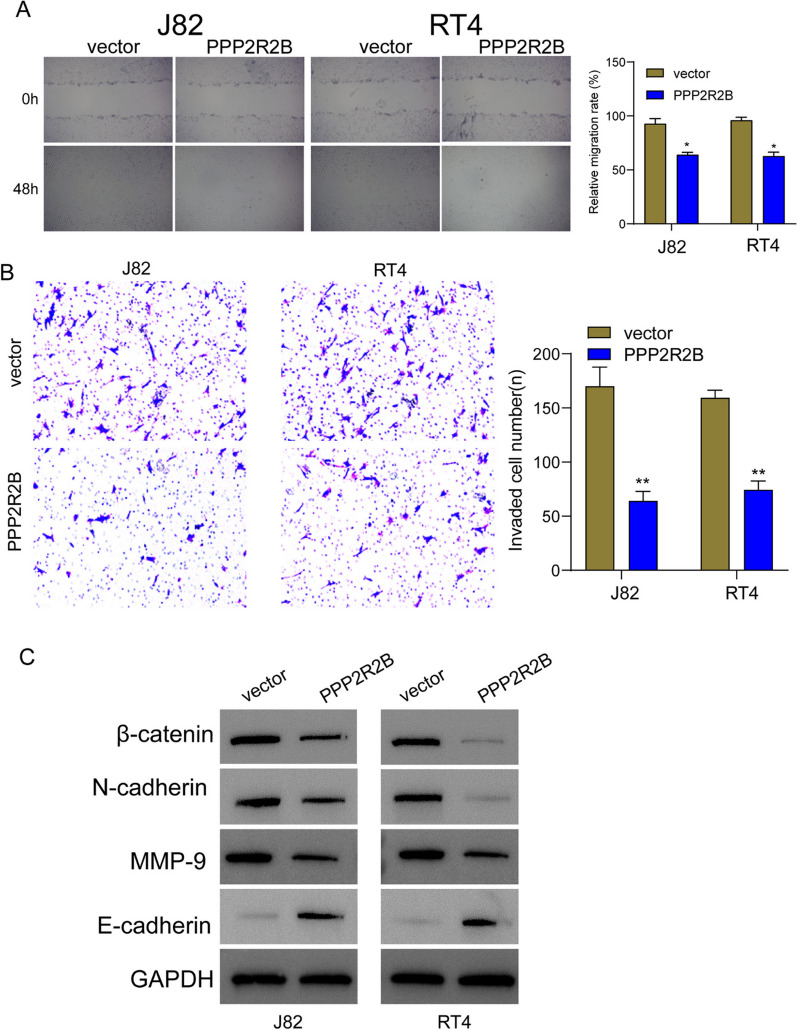


The correct Figure 9 is:
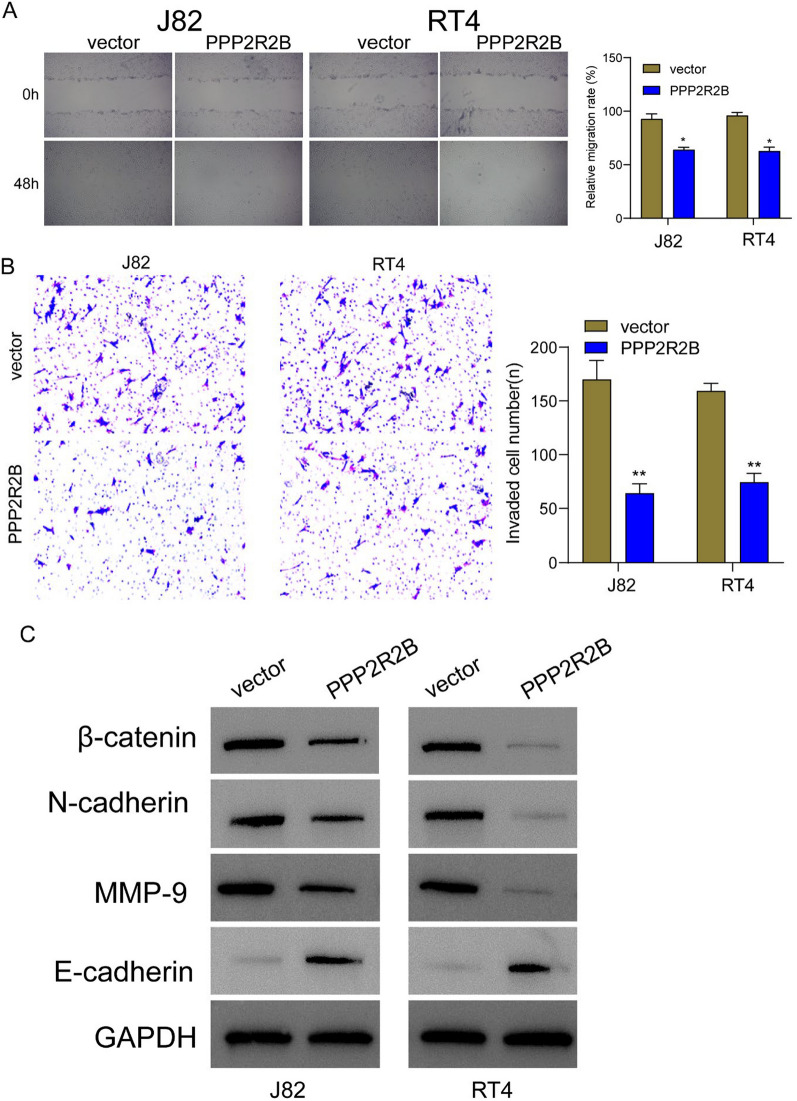


The original publication has been corrected.

## References

[CR1] 1. Shen and Kang (2024) Comprehensive analysis of mitochondria-related genes indicates that PPP2R2B is a novel biomarker and promotes the progression of bladder cancer via Wnt signaling pathway. Biology Direct 19:17 https://doi.org/10.1186/s13062-024-00461-6 10.1186/s13062-024-00461-6 PMC10898125 38409085

